# Big Data, Machine Learning, and Personalization in Health Systems: Ethical Issues and Emerging Trade-Offs

**DOI:** 10.1007/s11948-025-00552-1

**Published:** 2025-10-13

**Authors:** Stefano Canali, Alessandro Falcetta, Massimo Pavan, Manuel Roveri, Viola Schiaffonati

**Affiliations:** Department of Electronics, Information and Bioengineering, Politecnico di Milano, Milan, Italy

**Keywords:** Big data, Machine learning, AI ethics, Personalization, Health systems

## Abstract

The use of big data and machine learning has been discussed in an expanding literature, detailing concerns on ethical issues and societal implications. In this paper we focus on big data and machine learning in the context of health systems and with the specific purpose of personalization. Whilst personalization is considered very promising in this context, by focusing on concrete uses of personalized models for glucose monitoring and anomaly detection we identify issues that emerge with personalized models and show that personalization is not necessarily nor always a positive development. We argue that there is a new problem of trade-offs between the expected benefits of personalization and new and exacerbated issues – results that have serious implications for strategies of mitigation and ethical concerns on big data and machine learning.

## Introduction

The widespread use of big data and machine learning (ML) in various areas of contemporary societies has been accompanied by increasing concerns about ethical issues and societal implications. Among the wide range of application scenarios, these technologies are increasingly used in health systems, i.e. the organizations, institutions, and resources such as hospitals, insurance, or public health mandates that manage and implement healthcare (WHO, 2007; Frenk, 2010). Here, big data and ML are considered capable of providing effective and efficient solutions for monitoring, assistance, and care, but they can also raise significant ethical concerns, including concerns on autonomy, surveillance, exploitation, and profiling. In an expanding philosophical literature, these issues have been discussed in connection with broader challenges that these technologies bring to contemporary societies. Consider for instance privacy and the extent to which individual users have access and ownership of data (Hummel et al., 2020; Hummel & Braun, 2020). Health systems also present specific challenges, such as the blurring between public and private services, as private spaces such as the home are increasingly datafied and public-supported health systems are progressively privatized (Ishmaev, 2020). The extensive use of big data and ML can also lead to additional repurposing of personal health data for unforeseen and invasive inferences (Mittelstadt & Floridi, 2016; Fiske et al., 2022; Green et al., 2022).

There is a gap in this strand of the philosophical literature that we intend to fill in this paper: the ethical status of using big data and ML for personalization in health systems.

Personalization is among the perceived main strengths of big data and ML: the increasing availability of large data volumes on individual users enables the training of ML models on these personal data, in order to develop models that can detect specific personal features, aspects, and conditions of individual users. Personalization is already extensively employed, for instance in recommender systems. While the ethical status of personalization in these systems has been discussed (Airoldi, 2022; Akpinar et al., 2022), little work has looked at personalization through big data and ML in the context of health systems. This is surprising, because in the health context personalization is at the center of increasingly influential scientific and political frameworks such as precision and personalized medicine and related discussions (Prainsack, 2017, 2020; Green & Vogt, 2016; Green et al., 2023; Tabery, 2023).

Personalization is usually seen as an extremely positive approach with clear and consequential benefits for new health systems. However, our work shows that personalization is not necessarily nor always a positive development and there is a new problem of trade-offs between the expected benefits of personalization and new and exacerbated issues. In particular, we show that in health systems specific issues emerge with personalization – concept drift, burdens of data, and oversimplification – and have a significant impact on ethical concerns, including informed consent, data access, and quality of care. Discussing new and innovative technical approaches – Tiny Machine Learning and Homomorphic Encryption – we show that some of the negative effects of personalization we have identified can be mitigated, but limitations remain and further trade-offs arise. We conclude that health personalization through big data and ML is an approach that raises substantial trade-offs between expected benefits and negative effects – significant results that fill in the gaps in the ethical literature and have serious implications for strategies of mitigation.

To ground our work we build on a case-study of big data and ML for health systems that allows us to compare the use of predefined and personalized ML models. The case is based on Continuous Glucose Monitoring devices that detect anomalies for individuals interested in tracking biological parameters related to diabetes. Following current technological developments, we distinguish between scenarios where the analysis of glucose measurements is based on predefined or personalized models. Distinguishing between these technological scenarios in the case-study shows that some specific issues arise with personalization and put new and additional pressure on key ethical concerns.

The paper opens with a presentation of our case-study on Continuous Glucose Monitoring devices that collect big data on personal glucose levels and use ML to detect anomalies, through predefined and personalized models (*Continuous Glucose Monitoring and Personalization in Health Systems*). We approach it as an exemplary case of various crucial aspects of current big data collection practices and different modes of training and inference for ML models. In the section *Emerging Concerns from Personalization* we present our analysis of the significant negative effects of personalization, by focusing on three issues that emerge and are exacerbated with personalization: concept drift, which refers to scenarios where ML models can fail to detect changing relations between input and output and has implications for informed consent; new burdens of data, that emerge for users with personalization as more data have to be collected and tested, with effects on data control and privacy; and oversimplification, related to the computational demand of personalization, with significant impact on ethical issues related to the quality of healthcare services. Moving in the direction of mitigating those issues in the section *Mitigating Concerns from Personalization: Possibilities and Limitations of Privacy Preserving ML* we present innovative technical approaches that can be used to address and mitigate some of the concerns we have identified, with a particular focus on data privacy and control. However, our analysis shows the limitations of these approaches and the further trade-offs with other values and ethical considerations these create. We come to the conclusion that personalization should be seen at the center of trade-offs between expected benefits and negative consequences and should therefore be at the center of ethical discussions on big data and ML in this context.

## Continuous Glucose Monitoring and Personalization in Health Systems

Continuous Glucose Monitoring systems (CGMs) are devices that collect data on glucose levels. They are mostly used by diabetic patients who treat their condition with insulin and can benefit from the constant monitoring of their glucose levels to know when to take medication. We focus here on CGMs used to detect an anomaly in glucose levels and we distinguish two different scenarios where this function is executed, the first with a predefined model and the second with a personalized model. Whilst we do not refer to a device in particular that is available on the market, we ground our analysis on current use cases and innovative yet concrete technological trends that can be found in several devices, some of which are already on the market and others that are currently under development for CGM implementation (Hughes et al., 2023).

CGMs are an exemplary case for the current landscape of big data and ML for health systems and movements toward personalization. First, CGMs are exemplary of current practices of big data collection at the personal and individual level, thanks to wearable devices. Wearables are devices that are worn directly on our bodies and collect data on different aspects related to our health and are at the center of digital and mobile health initiatives (Sim, 2019; Canali et al., 2022; Friend et al., 2023). In a recent series published in the leading medical journal *New England Journal of Medicine* (Friend et al., 2023), one of the first published articles was dedicated to CGMs, presenting these devices as one of the most common types of wearable and digital health technologies (Hughes et al., 2023). Focusing on CGMs thus allows us to discuss a prominent example of the collection of big data on personal and individual conditions, that are considered necessary in the direction of personalized health systems. Second, CGMs show the current possibilities and exemplify the concrete application of ML for health systems. CGMs have modules that enable the connection to Cloud services or dedicated chips and neural engines to perform the training and inference of ML tasks on device. Devices such as CGMs provide a very significant and promising context for ML experimentation (Topol, 2023). Moreover, CGMs can show us the expected benefits and prospects of ML for health systems, where for instance ML models can be used to notify the user if, according to their current parameters, an anomalous situation is happening and there has been an unexpected spike in glucose levels in their blood. Therefore, focusing on CGMs allows us to analyze a concrete example of the current implementations of ML and discuss the prospects of developing models trained on personal glucose levels for personalized recommendations and intervention on individual health.

Our case-study in CGMs thus grounds our analysis in the rest of the paper on state of the art technology of big data and ML for health systems and works as a comparison between predefined and personalized scenarios. More specifically, we consider a case with CGMs using ML models to detect an anomaly in glucose levels, where anomaly detection is a classification task that is executed through a *training* and an *inference* phase. Our case represents ML models developed through *supervised learning*, where during the training phase ML models are trained to ‘learn’ the association between two variables in a dataset, i.e. glucose levels and their interpretation as anomalies or regularities. The goal is to detect similar associations when analyzing new data, e.g. detecting a state of anomaly in the glucose levels of a new user. The phase where ML models learn these associations is called *training* and the phase where anomalies are detected *inference*. While the inference phase happens always on data collected from the patient, the training phase can focus either on data coming from a large database, including medical records of many different people, or on the data of the patient directly. We talk about *predefined models* in the former case – when the ML model is trained on data of patients paid to record their data or data collected by health professionals for other research purposes. We instead refer to *personalized models* when the ML algorithm is trained only on data collected from the person who is monitored, for instance a dataset containing a week of recordings of glucose levels in the blood of the patient.

**Fig. 1:**
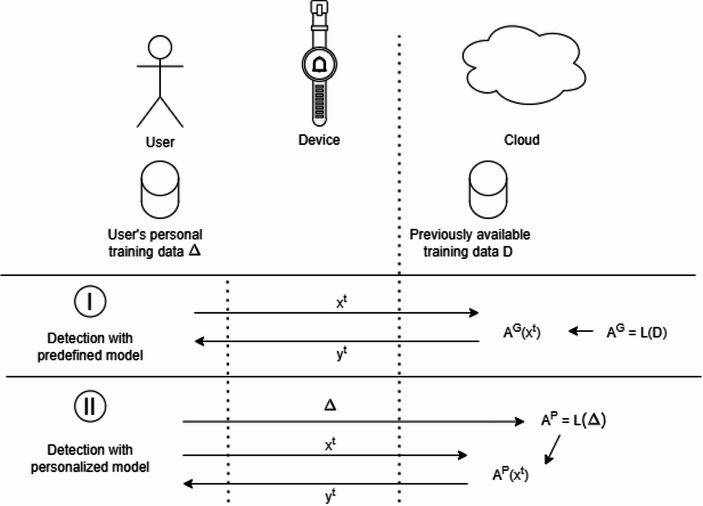
Summary of the types of data processing and models employed in the two scenarios of our case-study

The preparation and use of predefined and personalized models can be formalized in the following two scenarios for comparison (Fig. 1).

In Scenario I, a predefined model A^G^ is trained, using a learning procedure L, over the data D present in a large health database. After the training phase, A^G^ can be used to execute inference over the data x^t^ collected through the CGM device, and the result of this inference y^t^= A^G^(x^t^) is reported back to the user. In this scenario, the unlabeled data collected from the user are continuously sent to the Cloud during the regular usage of the device to perform inference.

In Scenario II, the one concerning the execution of personalized models, users are required first to collect a set of data for the training of the model. This set can be composed of recordings of the first usage of the device, for example, as well as the respective labels about whether one or more anomalies have happened during the data collection. This set of data, called Δ, is sent to the Cloud, and used to train a personalized model A^P^ through a learning procedure L. After this training phase, A^P^ can be used to execute inference over the data x^t^ collected through the CGM device, and the result of this inference y^t^= A^P^(x^t^) is reported back to the user. In this scenario, on top of the inference data, a set of supervised data needs to be collected too and sent to the Cloud.

These two scenarios follow recent Cloud-edge technological architectures (Milojicic, 2020), where big data collected by devices are moved to the Cloud for training and inference of ML models. The described approaches are currently widely adopted in the health domain to address tasks that require the continuous monitoring of patient health data (Aceto et al., 2020). Currently, Scenario I is widely more common in real-world applications, but the adoption of personalized ML models as in Scenario II for health monitoring is expected to be available on commercial devices.

## Emerging Concerns from Personalization

So far we have introduced and discussed two different scenarios where big data on glucose levels are collected and ML models are used to detect anomalies therein. On this basis, we can look at the comparison between predefined and personalized models from a philosophical point of view. As we will see, this comparison shows that new and exacerbated issues emerge with personalization, which have a significant impact on ethical concerns. In particular, we show that issues of concept drift, burdens of data, and oversimplification have an impact on key ethical concerns that have been discussed in the literature and show that it is crucial to see personalization and its emerging trade-offs.

### Concept Drift

The issue known as *concept drift* in ML research (Ditzler et al., 2015) describes scenarios where the relation between input and output changes over time, with implications on the statistical behavior of the data generating process (Roveri, 2019; Disabato & Roveri, 2021). In other words, the meaning of the input data that the model was trained on might change over time and do so significantly; the model might not capture this change and therefore its predictions will decrease in accuracy. Consider in our case changes in the user’s behavior and lifestyle, which might affect the statistical distribution of glucose levels to the point that, for instance when the user is older, what count as standard or anomalous glucose levels might be very different from the initial training dataset. As a result of this issue, models might not be able to adapt to the change and drift of the data, with a possibly dramatic decrease in their accuracy over time.

What we can show with our comparison between predefined and personalized ML is that this behavior becomes particularly significant with personalization – the increasing reliance on personalization does not necessarily mean that models will adapt properly to the personal features and changes in the conditions of the users that can happen over time. As individuals change and so does their condition, personalization can lead to degrading predictive performance and poor results that were unexpected when users started relying on the device for anomaly detection. This could lead, in turn, to inconsistent and unpredictable model behavior, even at the level of data that could normally be analyzed with predefined models. As a result, with personalized models concept drift emerges as even more problematic. Concept drift is a known issue in the literature on ML models, but its emergence and role with personalization are significant from a philosophical viewpoint too – concept drift puts new and additional pressure on key ethical issues.

Clearly, the fact that data can drift and as a result models can have decreasing accuracy presents new trade-offs between accuracy and ethical principles of safety, beneficence, and non-maleficence. But here we want to highlight how concept drift puts new and increasing pressure also on other ethical considerations, such as those looking at consent.

With these forms of concept drift we can identify new and significant constraints on how consent can be given: a lack of understanding and control over the results of personalization makes it very difficult to give informed consent to results that might be unexpected, unpredictable, and not explainable. The issue is particularly problematic because results from personalized anomaly detection can limit individual autonomy.^1^ Choices of which food to consume, whether to get insulin, which activities to engage with – these are clearly informed and limited by the anomaly detection provided by the models we discuss. The problem here is that obstacles to autonomy may be stronger when indications are personally tailored to the individual patient and user and this, coupled with limitations connected to opacity and explainability, poses significant and new constraints on consent.^2^

### Burdens of Data

A second reason to see personalization as something concerning and with ethical implications is due to the new *burdens of data* that personalization can create (Lomborg et al., 2020, p. 8).

Significantly more data have to be collected for the personalization of ML models. In our personalization Scenario II, personal data about the glucose levels of the individual user are collected, not just for performing new inferences but crucially also during the training phase. This can have a significant impact on a series of issues connected to data protection and privacy, that are more significant as a result of personalization. This is particularly clear when we consider that personalization crucially relies on Cloud services for training and inference, and these have been discussed in the philosophical literature as particularly problematic obstacles to data property and data sovereignty (Hummel et al., 2020; Hummel & Braun, 2020). As more and more personal data are shared and analyzed on Cloud services, concerns about potential and known cases of re-use for additional and unwanted uses emerge (e.g. profiling, advertising, targeting, etc.).

The problem here is that with personalization these concerns become even more expansive and pose new trade-offs between expected benefits and new burdens. In order to personalize models, as we have seen, we have an increasing use of personal health data – data about personal glucose levels are not used only at the inference step, such as when using predefined models, but also at the training level. Moreover, these data also need to be acquired with specific labels, that indicate whether the personal glucose levels are anomalous and problematic for the specific user of the models. This creates a situation where more personal data and sensitive information about the health status and conditions of individuals have to be collected and shared, presenting new concerns about possible and unwanted uses.

Hence, personalized models put additional pressure on ethical considerations about data access and privacy. But there is more – personalization can lead to new burdens related to responsibility. Users might want to share monitoring data and results with carers and health professionals, but since the ML model in the personalized Scenario II will be personalized and thus unique to the user, this might be more difficult and less explainable for users, carers, and health professionals. Personalized models might not be accessible for device manufacturers, which can prevent them from ensuring specific levels of quality without collecting feedback from the user. In this way, the user has to be more actively responsible for both collecting the data that the model needs (and thus responsible also for the quality of those data) and for checking the performance of the model, in order to establish its quality and, if more data are needed, to further fine-tune it. This poses new problems of responsibility on informal carers and users themselves, in connection with discussions on responsibility and related gaps as key issues in the ethics of ML (see e.g. work by Lang, Nyholm, Blumenthal‑Barby, 2023).

### Oversimplification

A further set of considerations that we want to show as emerging as a result of personalization is connected to the issue of *oversimplification*. In our case-study, the use of personalized models can be seen as a way of opening up the possibility of including other contextual information (e.g. when GCM provides glucose data to electronic health records or other Cloud services). However, this can be at the expense of the complexity of parameters for measurement and automation. Whilst in our case-study the parameters used to collect data on glucose monitoring and detect anomalies therein are relatively simple, concerns on how much these have to be simplified, with possibly harmful effects for users, are more critical with personalized models.^3^

This is connected to a specific aspect of ML, its computational demand, which is extensive and becomes particularly substantial with personalization. Compared with the predefined Scenario I of our case-study, personalized models can be more computationally demanding because of the need to collect and label new data and perform both training and inference on these data. The problem here is that as a result personalization of ML models can require models that are simpler than pre-defined models. These limitations, in turn, raise concerns about the extent to which measurement has to be simplified for training and personalization to be feasible and how much is “lost” as a result of oversimplification. Personalizing the detection of anomalies at an individual level, in other words, can amplify the risk of simplifying the ways in which anomalies are detected and measured. By oversimplification here we refer both to the fact that ML systems are usually based on data collection of simpler health parameters such as glucose levels, heart rate, step-counting and of course other and more complex aspects that are often not registered. Yet oversimplification is also implied in the fact that personalized models need to be simpler and easier to execute on the basis of more restricted and simpler computational capabilities.

Oversimplification here is particularly problematic from an ethical point of view on trade-offs with the quality of healthcare services. Concerns about the risks of decontextualized and quality levels of healthcare services have been expressed in the literature on health systems that increasingly rely on and make use of big data and ML, for instance in relation to the fact that this approach can “result in ignorance of aspects of the patient’s health that cannot easily be monitored” (Mittelstadt, 2017, p. 167). Our analysis, grounded in the differences between the two technological scenarios, shows that personalized ML models create more pressure because of oversimplification. While supposedly ever-growing computational capabilities of ML have the potential to build more integrated and holistic understandings of health, we identify a growing risk in relying on overly simplified if not reductive parameters – an issue that is particularly significant when personalized models are employed.

## Mitigating Concerns from Personalization: Possibilities and Limitations of Privacy Preserving ML

In the analysis of our case-study on predefined and personalized ML models we have shown that new and exacerbated issues can emerge with personalization, and these have an impact on several ethical concerns discussed in the literature. This frames personalization as a new and emerging area of concern for philosophical discussions, that need to focus on personalization as a specific set of ethical considerations and on the specific effects that personalized systems create in the context of health systems.

On the basis of these results we now discuss the need for mitigation strategies on the issues that we have identified. In particular, we show that some of the most advanced approaches and technologies available in the field focus on privacy protection and preservation and can be used to mitigate some of the concerns we have identified. However, we highlight that these approaches have crucial limitations and further show that personalization leads to the emergence of additional trade-offs between expected benefits and negative consequences.

### Homomorphic Encryption

Several privacy-enhancing technologies have been proposed in recent years. Among them, differential privacy (Dwork, 2006) works by injecting noise into data, making it harder to distinguish between two data points. K-Anonymity (Dalenius, 1986), on the other hand, obfuscates or generalizes certain parts of the data to achieve the same goal. However, the only approach that allows encrypted data to be processed without any data perturbation is Homomorphic Encryption (HE). HE is a special type of encryption that enables certain operations to be performed on encrypted data without requiring prior decryption (Falcetta & Roveri, 2022). The output of an operation on two ciphertexts remains encrypted. In recent years, the intersection of HE and ML has led to the growth of Privacy-Preserving ML with HE (PP-ML), a novel research area focused on developing ML models capable of performing inferences on encrypted inputs. While training ML models on encrypted datasets is more challenging (Podschwadt et al., 2022), it remains an active and promising research area.

In the scenarios for GCM proposed in previous sections, PP-ML can be applied to protect user data privacy in both cases. If a predefined model is used, such as in Scenario I, only the data for inference needs to be encrypted before being sent to the Cloud. There, the provider can run a PP-ML model specifically modified to process encrypted inputs. In the case of a personalized model of Scenario II, the entire training dataset could be encrypted before being sent to the Cloud, where specialized encrypted learning algorithms will be used to train an encrypted model on the encrypted dataset. This model is later be used for encrypted inference.

**Fig. 2:**
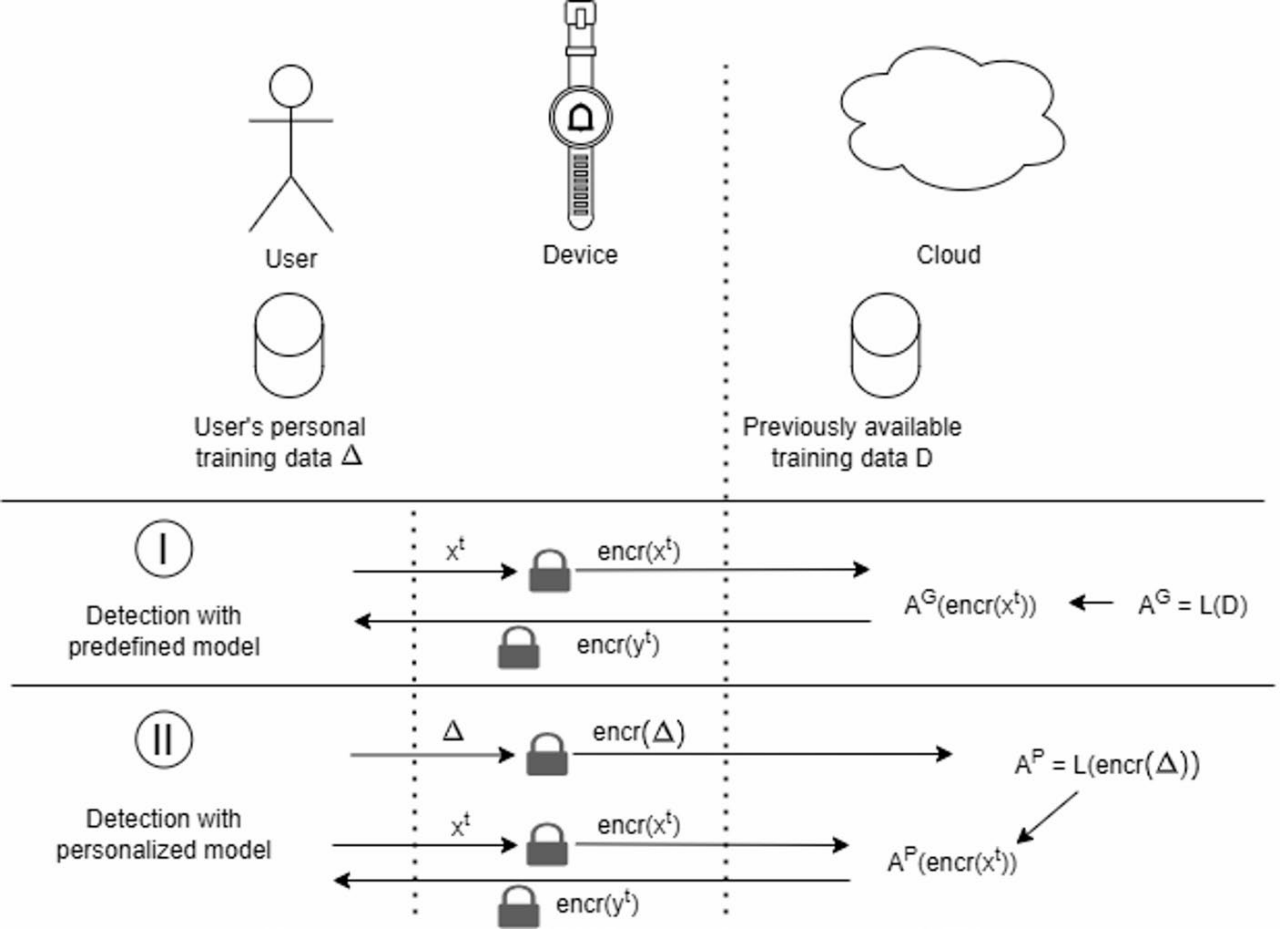
Use of homomorphic encryption in the two scenarios of our case-study

More specifically, as in Fig. 2 the formalization slightly changes with the application of HE: in the predefined Scenario I the model A^G^ is still executed in the Cloud and trained on a health dataset D, but the data it is working on, encr(x^t^), are previously encrypted on the device. The results of the computation encr(y^t^) are still encrypted, and can be decrypted only on the device. In the case of the personalized model, not only the data x^t^ are encrypted, but also the dataset Δ is encrypted, before being used by the encrypted learning mechanism to create the personalized model A^P^.

### Tiny Machine Learning

Another possible technical approach to mitigate some of the issues we have identified is to go further in the direction of executing all operations on device. This is the direction of techniques such as Tiny Machine Learning (TinyML), a novel research area aiming at designing ML models that can be executed on tiny devices, such as IoT and wearable devices. The research in this area is making significant steps in the field of frameworks (David et al., 2021), algorithms (Gholami et al., 2021) and approximation mechanisms (Liu et al., 2020), models and learning paradigms (Alippi et al., 2018).

Advances in this line of research allow ML models to overcome constraints on computation, memory, and energy consumption, hence paving the way for a pervasive diffusion of TinyML applications in everyday life (e.g. smart homes and buildings, smart cars, e-health, industry 4.0). More generally, while all commercial applications of TinyML work in a train-then-deploy fashion (i.e. the model is trained on the Cloud or on a desktop PC and then deployed on the device), significant research is currently under development in the field of on-device training, with the goal of enabling the training of TinyML algorithms directly on the units that will employ them (Pavan et al., 2023; Rajapakse et al., 2023).

Although in different ways from HE, TinyML is seen as a way to protect and preserve individual privacy. In Scenario II where a personalized model is developed and deployed, using a TinyML approach implies moving both training and inference to the device. This also implies that personal data will not leave the personal device of the user, thus protecting unwanted uses of the same data.

**Fig. 3:**
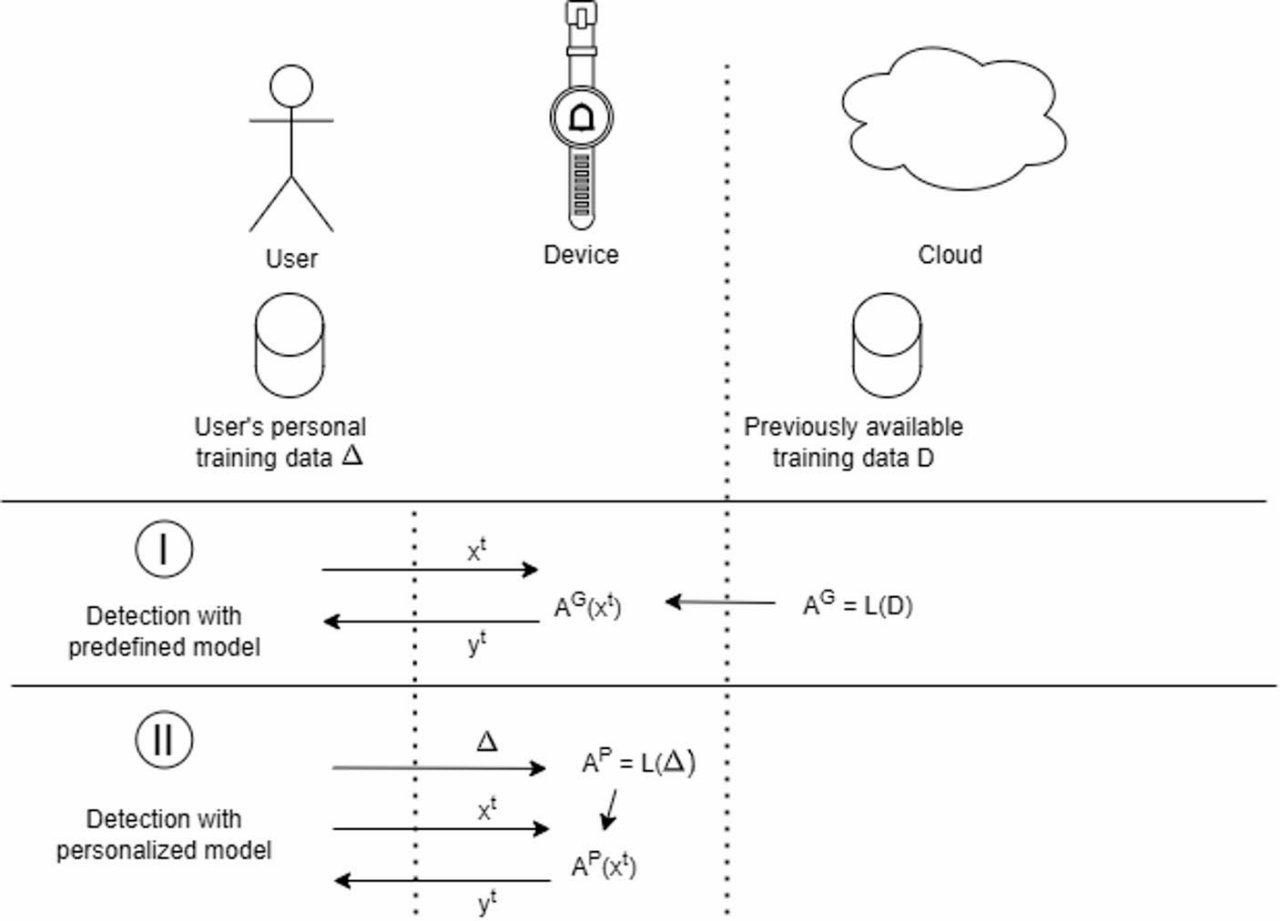
Use of TinyML in the two scenarios of our case-study

In our case-study, as in Fig. 3, the formalization slightly changes if we move the model on-device using TinyML: in the predefined Scenario I the model A^G^ is still trained in the Cloud on a health dataset D, but after this phase A^G^ is deployed on the device, where the inference on the data of the patient will be executed. In the case of a personalized model, not only the inference phase is executed on the device, but also the learning phase happens on-device through the usage of an optimized learning mechanism L.

### The limitations of Privacy-Preserving Solutions

Technical approaches that focus on privacy, such as HE and TinyML can be used to mitigate some of the issues we have identified in the previous section. HE, by encrypting the data on the device, makes it basically impossible for the provider of the service to violate the privacy of the user, but also to maintain forms of data control. TinyML, by moving the computation completely on the devices, and performing the data analysis directly there, achieves the same advantages as HE on the topics of privacy and control.

However, these approaches also introduce additional challenges and trade-offs that have to be considered. For instance, some scholars have recently raised concerns about the extensive use of TinyML models in relation to the propagation of different types of bias, in particular what they call “​​reliability bias” (Hutiri et al., 2023). In the context of our case-study the control over the data that these technologies guarantee does not necessarily correspond to an improvement in the access of the data. Considering that in both approaches the (decrypted) data are available only on the device and that these types of devices usually can rely on limited amounts of memory, the volume of data that will be accessible to the patient will inevitably be limited. Furthermore, the on-device location of the data can further limit possibilities of sharing with third parties (such as, for example, medical staff), since the restrictive human-computer interfaces usually present in these devices can make the distribution of these data more difficult for non-technical users.

While HE and TinyML limit the access to the data of the final user to the service provider, they limit also access to the model itself. This can make it very difficult for these models to be properly validated, periodically checked, or corrected. In this context, the only way to ensure the correct working of the personalized ML models is by providing correct data to the model and monitoring the performance of the algorithm – tasks that can be performed only by users and patients. By shifting the responsibility for the correct functioning of the algorithm onto the patient, our aforementioned concerns on burdens of data and oversimplification can be even more significant. Furthermore, TinyML and HE models can work only with the data provided by the device itself. This limits the possibility for further cross-analysis of the data in conjunction with any other data collected by different sensors or the considerations of other conditions in the data analysis, enhancing the concerns on decontextualization of the health data.

Finally, both methodologies have to deal with strong technical limitations. With TinyML, these limitations are related to the wearable hardware where the ML model runs, which is designed to consume low power and thus is limited in terms of computational capabilities and memory. With HE, limitations are connected to the encryption of the data and the models, which causes the requirements of the algorithms to grow exponentially. both in terms of memory and computational capabilities. These limitations contrast with the requirements of personalized models – when running also the training phase, on top of the inference, the limitations get even more restricting. While in principle it is possible to simplify the algorithms (and reduce the resolution of the input data), these limitations create additional concerns on the extent to which measurement has to be oversimplified for training and personalization to be feasible on device and how much is lost as a result of oversimplification. By enabling privacy-preserving personalization, in other words, there is the risk of overly simplifying the models, in a way that makes personalization less effective than using a pre-defined model.

## Conclusion

The promises of big data and ML seem to invest medicine at all its levels, and personalization is at the center of many of them (Topol, 2023). Personalization is also one of the potentially more tangible prospects of big data and ML for health and among the most beneficial – as personalization becomes increasingly available in commercial technologies such as wearables, we have seen that it can be used in concrete cases with almost immediate consequences for individual users (Cangardel & Volgina, 2023). The appeal is clear here: Who would not want health, such a fundamental aspect of our lives, treated with individually tailored methods that do justice to our unique features? And why not receive all this thanks to what currently is considered most innovative technology?

In contrast with this narrative, concrete cases of application of the use of ML for personalization show several concerns related to key ethical and epistemic values. Together, the issues we have identified in the central sections of the paper show that personalization should be seen as a technological approach that comes with possible benefits as well as concerns and is at the center of trade-offs in this direction. Namely, as much as the use of personalized models for detecting anomalous glucose levels can be beneficial to the user thanks to increasing accuracy and personalized recommendations, we have seen that new issues emerge, are exacerbated, and have an impact on ethical concerns. This creates a situation where we need to understand how to balance the trade-offs that come from the expected benefits of personalization (e.g. accuracy) and possible issues (e.g. concept drift). Similarly, available mitigation strategies tend to focus on one set of concerns and can be beneficial in this direction (e.g. privacy), but might introduce and exacerbate other issues (e.g. responsibility). Again, this is a situation with significant trade-offs, where we may need to take difficult decisions about what we consider more important and worth addressing (e.g. privacy over responsibility).

Seeing personalization as an approach that raises significant trade-offs is a new result that we have shown in this paper. We see our results on the trade-offs of personalization as a starting point for developing an understanding of how to balance and weigh trade-offs between expected benefits and possible issues. More concretely, for example, this understanding leads to discussions on where and how to implement personalization and on the possible need to limit personalization in specific contexts. We need to question and understand whether personalization is good enough and entertain the option of limiting some forms of personalization through ML. For instance, in other work (Falcetta et al., 2023) we have developed an algorithmic approach that tries to mitigate the negative effects of personalization by backtracking and limiting personalization. This can be done by constraining the training procedures of ML models, in a way that personalized models satisfy pre-defined boundaries. This may, in general, decrease the final performances of such models, for instance the detection of anomalies during glucose monitoring, but on the other hand would let the training required for personalization proceed in a “guided” way. By limiting the search space of personalized solutions, models can provide more guarantees to prevent harm to the final user and respect of specific epistemic and ethical values (e.g. representativity, quality, non-maleficence, beneficence). Also, with this approach clearly not all the issues we have identified in previous sections are solved and the approach might not be as effective as a fully personalized approach. In other words, in a context of trade-offs, limiting personalization is a choice that privileges some considerations and values over others, and goes in the direction of the need to address trade-offs and take specific choices as to which values and principles should be preferred in developing ML models and personalization.

Therefore, personalization creates new trade-offs between expected benefits and new and exacerbated issues that arise as a result. These results are consequential for strategies of mitigation for the ethical concerns on big data and ML – as we have shown these will need to be renovated in light of innovative technical approaches in the field of ML, particularly privacy-preserving techniques, and considering implications for policy and ethical approaches. We see this analysis as a starting point for more work to make sense of the effects of personalization as an increasingly technological possibility to be applied in the health context, where philosophical and technical approaches can be integrated to encourage beneficial results and avoid harmful applications.

## Footnotes

1 Autonomy is of course one of the central principles of bioethics (Estes, 2022) and as such is also one of the most discussed ethical concerns in the ethical literature (Heinrichs, 2021; Chiodo, 2022), particularly in the health context (Essén, 2008; Remmers, 2010; Blasimme & Vayena, 2016).

2 As helpfully suggested by an anonymous referee, on top of our considerations on consent, concept drift can also be a concern for the safety of the actions recommended by the device.

3 For more on simplification and its relation to reductionism see also Angelucci et al. (2023).
